# Fabrication and Physical Evaluation of Gelatin-Coated Carbonate Apatite Foam

**DOI:** 10.3390/ma9090711

**Published:** 2016-08-23

**Authors:** Kanae Hara, Kenji Fujisawa, Hirokazu Nagai, Natsumi Takamaru, Go Ohe, Kanji Tsuru, Kunio Ishikawa, Youji Miyamoto

**Affiliations:** 1 Department of Oral Surgery, Institute of Biomedical Sciences, Tokushima University Graduate School, 3-18-15 Kuramotocho, Tokushima 770-8504, Japan; kanahara@tokushima-u.ac.jp (K.H.); hnagai@tokushima-u.ac.jp (H.N.); takamaru@tokushima-u.ac.jp (N.T.); go.ohe@tokushima-u.ac.jp (G.O.); miyamoto@tokushima-u.ac.jp (Y.M.); 2 Department of Biomaterials, Faculty of Dental Science, Kyushu University, 3-1-1 Maidashi, Higashi-ku, Fukuoka 812-8582, Japan; tsuru@dent.kyusyu-u.ac.jp (K.T.); ishikawa@dent.kyusyu-u.ac.jp (K.I.)

**Keywords:** Carbonate apatite, foam, interconnected porous structure, gelatin, heat treatment

## Abstract

Carbonate apatite (CO_3_Ap) foam has gained much attention in recent years because of its ability to rapidly replace bone. However, its mechanical strength is extremely low for clinical use. In this study, to understand the potential of gelatin-reinforced CO_3_Ap foam for bone replacement, CO_3_Ap foam was reinforced with gelatin and the resulting physical characteristics were evaluated. The mechanical strength increased significantly with the gelatin reinforcement. The compressive strength of gelatin-free CO_3_Ap foam was 74 kPa whereas that of the gelatin-reinforced CO_3_Ap foam, fabricated using 30 mass % gelatin solution, was approximately 3 MPa. Heat treatment for crosslinking gelatin had little effect on the mechanical strength of the foam. The gelatin-reinforced foam did not maintain its shape when immersed in a saline solution as this promoted swelling of the gelatin; however, in the same conditions, the heat-treated gelatin-reinforced foam proved to be stable. It is concluded, therefore, that heat treatment is the key to the fabrication of stable gelatin-reinforced CO_3_Ap foam.

## 1. Introduction

Carbonate apatite (CO_3_Ap: Ca_10-*a*_(PO_4_)_6-*b*_(CO_3_)*_c_*(OH)_2-*d*_) foam is a good candidate which can be used in bone replacement, because it has the same inorganic composition and fully interconnected porous structure as cancellous bone. It is fabricated by compositional transformation, based on a dissolution-precipitation reaction using α-tricalcium phosphate (α-TCP: Ca_3_(PO_4_)_2_) foam as the precursor [1,2,3,4]. Unfortunately, its mechanical strength is too low for its clinical application. This low mechanical strength is thought to be caused by its high porosity and the absence of organic material. Collagen plays an important role in the elastic properties of bone. In fact, osteogenesis imperfecta, or Lobstein syndrome, is known to be caused by a deficiency in type I collagen [5,6]. Recently, a poly (L) lactic acid glycolic acid (PLGA) copolymer and ε-caprolactone were reported to be effective in CO_3_Ap foam reinforcement [7,8,9,10]. In both cases, the mechanical strength of the foam was improved significantly by coating it with these materials. Although both polymers are classified as bioresorbable, the tissue response to them is relatively poor [7,8,9,10].

Gelatin is obtained by thermal denaturation or physical and chemical degradation of collagen through the breaking of the triple-helix structure into random coils [11]. When compared with collagen, gelatin does not express antigenicity under physiological conditions; it is completely resorbable in vivo, and its physicochemical properties can be suitably modulated. Furthermore, it is much cheaper and easier to obtain in concentrated solutions [12]. Because of its biodegradability and cytocompatibility [13,14], gelatin is clinically proven as a temporary defect filler and wound dressing. In addition, it is also useful for porous calcium phosphate material [15]. Gelatin is also known to exhibit good tissue response and has a long history of clinical use [16,17]. Therefore, the aim of this present study is to fabricate and evaluate the physical properties of gelatin-reinforced CO_3_Ap foam as an initial step towards its clinical use as an artificial bone substitute.

## 2. Materials and Methods

### 2.1. Preparation of Gelatin-Free CO_3_Ap Foam

CO_3_Ap foam was prepared by compositional transformation, based on the dissolution-precipitation reaction, as described previously [1,4]. In brief, polyurethane foam (HR-20D, Bridgestone Corp., Tokyo, Japan) with a fully interconnected porous structure, similar to cancellous bone, was used as a template. The polyurethane foam was dipped into a slurry of calcium carbonate (CaCO_3_, Wako Chemicals, Tokyo, Japan) and dicalcium phosphate dihydrate (CaHPO·2H_2_O, Wako Chemicals) with a Ca/P molar ratio of 1.5 to coat the foam’s struts with the powder. The powder-coated polyurethane foam was then heated in an electric furnace (SBV1515D, Motoyama, Osaka, Japan) at 1500 °C for 5 h. During the sintering process, α-TCP was foamed as the polyurethane foam burned off. The resulting α-TCP foam, which exhibited the same structure as the polyurethane foam, was then immersed in a 4 M ammonium carbonate ((NH_4_)_2_CO_3_, Wako Chemicals) solution where the compositional conversion from α-TCP to CO_3_Ap occurred while the macroscopic structure was maintained.

### 2.2. Reinforcement of CO_3_Ap Foam with Gelatin

Gelatin-free CO_3_Ap foam was immersed in an aqueous solution of 10, 20, and 30 mass % gelatin (G-2707P, Nitta Gelatin Inc., Osaka, Japan) at 60 °C for 1 h. The foam was then removed from the gelatin solution and any excess gelatin eliminated by air blowing. The foam was dried in a vacuum oven (AVO-250N, As One, Osaka, Japan) at 60 °C overnight. To cross-link the gelatin, some specimens were further heated, under vacuum, at 155 °C for 4 h [18].

In this paper, the concentration of the gelatin solution and presence or absence of heat treatment for crosslinking is stated in the parenthesis. For example, original or unreinforced CO_3_Ap foam without heat treatment was denoted as CO_3_Ap foam (0, none); whereas CO_3_Ap foam reinforced with 10 mass % gelatin and heat-treated for crosslinking is denoted as CO_3_Ap foam (10, HT). The amount of gelatin in forms was measured by weighting of the foams before and after gelatin was coated.

### 2.3. Scanning Electron Microscopy Observation

After sputter coating with gold-palladium, the microstructures of the specimens was examined using a scanning electron microscopy (SEM) (S-3400N, Hitachi High-Technologies Corp., Tokyo, Japan) at an accelerating voltage of 15 kV.

### 2.4. X-ray Diffraction Analysis

The composition of the specimens was determined using an X-ray diffractometer (XRD: D8 Advance, Bruker AXS GmbH, Karlsruhe, Germany) operated at 40 kV and 40 mA. The diffraction angle was continuously scanned, from 10° to 60°, in 2θ at a scanning rate of 2°/min.

### 2.5. Fourier-Transformed Infrared Spectroscopy

Fourier transformed infrared (FT-IR) spectroscopy, over a 370–7800 cm^−1^ wave number range, was performed with as FT-IR spectrometer (Spectrum 2000LX; Perkin-Elmer Co. Ltd., Waltham, MA, USA).

### 2.6. Mechanical Strength Evaluation

The mechanical strength of the specimens was evaluated in terms of the compressive strength. After measuring the dimension of the foam using a digital micrometer (IP65, Mitsutoyo, Kanagawa, Japan), each specimen was positioned parallel to the floor and crushed vertically at a crosshead speed of 1 mm/min using a table-mounted universal testing machine (Autograph AGS-J, Shimadzu, Kyoto, Japan). The compressive strength value was determined from the average results of at least five specimens. For statistical analysis, one-way factorial ANOVA and Fisher’s PLSD method as a post-hoc test were performed using the Stat View 4.02 software (Abacus Concepts Inc., Berkeley, CA, USA) at a significance level of 5%.

### 2.7. Stability of CO_3_Ap Foam in the Saline Solution

The stability of the CO_3_Ap foam when exposed to body fluids was evaluated by immersion in a saline solution at 37 °C for 24 h. A photograph was taken to evaluate the stability.

## 3. Results

**Figure 1:**
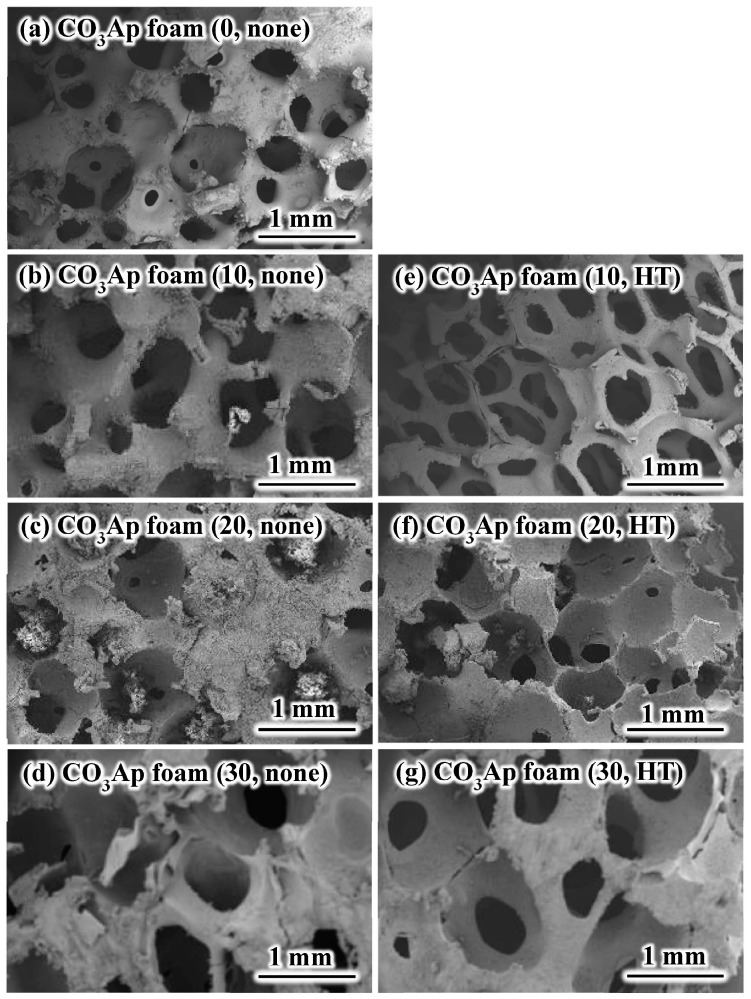
Typical scanning electron microscopy images of the CO_3_Ap foams: (**a**) CO_3_Ap foam (0, none); (**b**) CO_3_Ap foam (10, none); (**c**) CO_3_Ap foam (20, none); (**d**) CO_3_Ap foam (30, none); (**e**) CO_3_Ap foam (10, HT); (**f**) CO_3_Ap foam (20, HT); and (**g**) CO_3_Ap foam (30, HT).

Figure 1 presents typical SEM images of the CO_3_Ap foam with and without gelatin reinforcement, and with and without heat treatment. The pore size was approximately 300–1000 μm. Interconnected porous structures similar to cancellous bone were observed regardless of the presence or absence of the gelatin reinforcement or the solution concentration. No significant difference was observed between the presence and absence of heat treatment.

**Figure 2:**
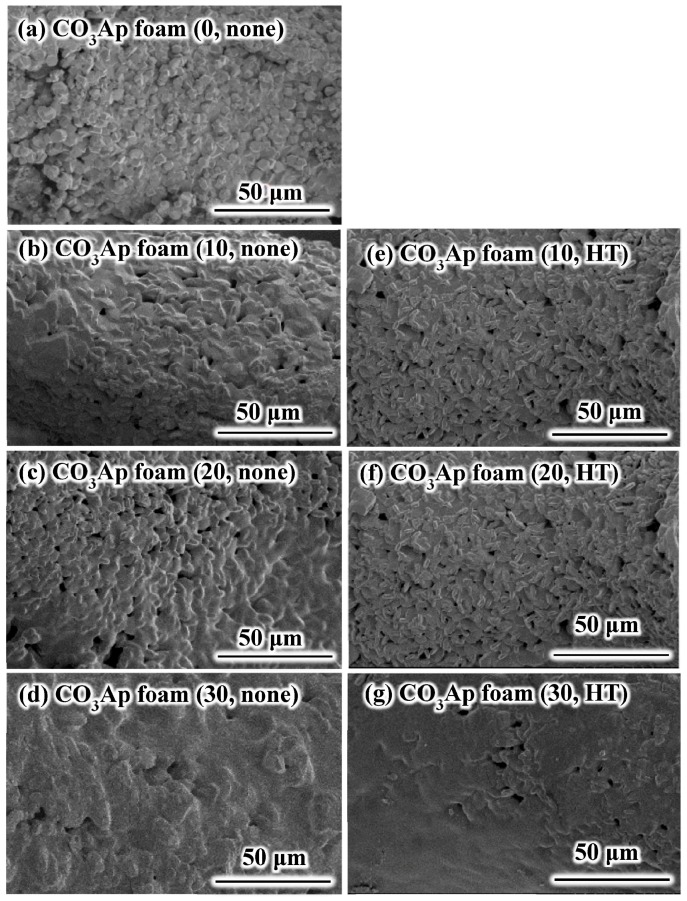
Typical scanning electron microscopy images of higher magnification of the CO_3_Ap foam strut: (**a**) CO_3_Ap foam (0, none); (**b**) CO_3_Ap foam (10, none); (**c**) CO_3_Ap foam (20, none); (**d**) CO_3_Ap foam (30, none); (**e**) CO_3_Ap foam (10, HT); (**f**) CO_3_Ap foam (20, HT); and (**g**) CO_3_Ap foam (30, HT).

Figure 2 presents the SEM images of the CO_3_Ap foam struts. Entangled crystals were observed at the surface of the CO_3_Ap foam strut (0, none) (Figure 2a). Gelatin covered the surface of the entangled crystals when the foam was reinforced. Although the amount of the gelatin coating the strut surfaces increased with the increasing solution concentration, no significant difference was observed in the presence or absence of heat treatment.

The amount of gelatin of the foam (20 mm × 10 mm × 10 mm) was 0.159 ± 0.015 g when immersed in 10 mass % gelatin solution, and 0.248 ± 0.006 g in 20%, and 0.426 ± 0.108 g in 30%.

**Figure 3:**
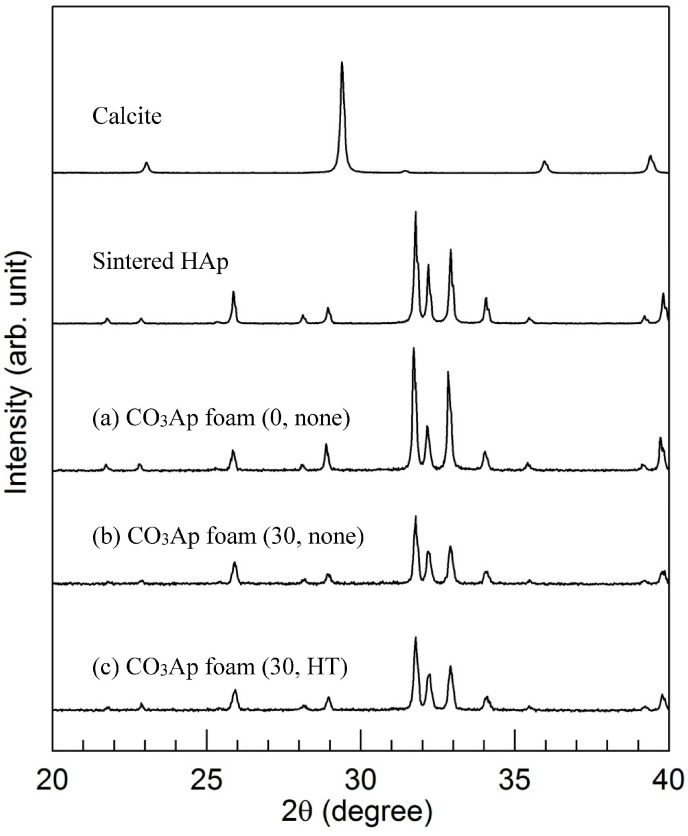
X-ray diffraction patterns of calcite, sintered HAp and CO_3_Ap foams: (**a**) CO_3_Ap foam (0, none); (**b**) CO_3_Ap foam (30, none); and (**c**) CO_3_Ap foam (30, HT).

Figure 3 presents XRD patterns of calcite, sintered HAp and CO_3_Ap foams: (a) gelatin-free CO_3_Ap foam; (b) 30 mass % gelatin-coated CO_3_Ap foam without heat treatment; and (c) 30 mass % gelatin-coated CO_3_Ap foam with heat treatment. All the specimens exhibited typical apatitic patterns, indicating the stability of the CO_3_Ap foam against the gelatin coating and heat treatment.

**Figure 4:**
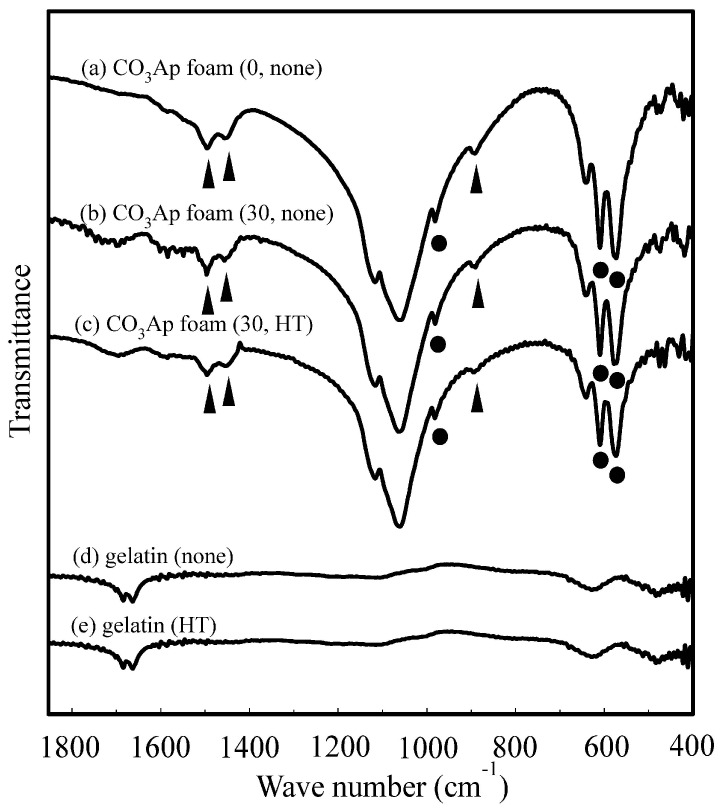
Fourier-transform infrared spectroscopy spectra of CO_3_Ap foams: (**a**) CO_3_Ap foam (0, none); (**b**) CO_3_Ap foam (30, none); (**c**) CO_3_Ap foam (30, HT); (**d**) gelatin without heat treatment; and (**e**) gelatin with heat treatment at 155 °C under vacuum for 4 h. Triangles; correspond to B-type CO_3_Ap peaks (1455, 1410 and 875 cm^−1^), Black circles; correspond to phosphate bands peaks (980–1100 and 560–600 cm^−1^).

Figure 4 presents the FT-IR spectra of (a) gelatin-free CO_3_Ap foam and the CO_3_Ap foam; (b) without and (c) with heat treatment after reinforcement with the 30 mass % gelatin solution. FT-IR spectra of (d) gelatin without heat treatment and (e) gelatin with heat treatment are also presented for comparison. Typical peaks of B-type CO_3_Ap were observed at approximately 1410, 1455, and 875 cm^−1^, along with peaks corresponding to phosphate bands at 980–1100 and 560–600 cm^−1^ [19]. These results indicated that the CO_3_Ap foam formed in the present study was a B-type CO_3_Ap, where the PO_4_^3−^ lattice site was substituted by CO_3_^2−^, similar to the bone mineral apatite. In addition, the CO_3_^2−^ content in the apatitic structure was 3.2 ± 0.7 mass %, calculated using the method of Featherstone et al. [20]. The presence of gelatin did not result in a sharp peak; however, broad peaks at approximately 560–600 cm^−1^ were observed for the gelatin-coated CO_3_Ap foam. No significant difference was observed before and after the heat treatment.

**Figure 5:**
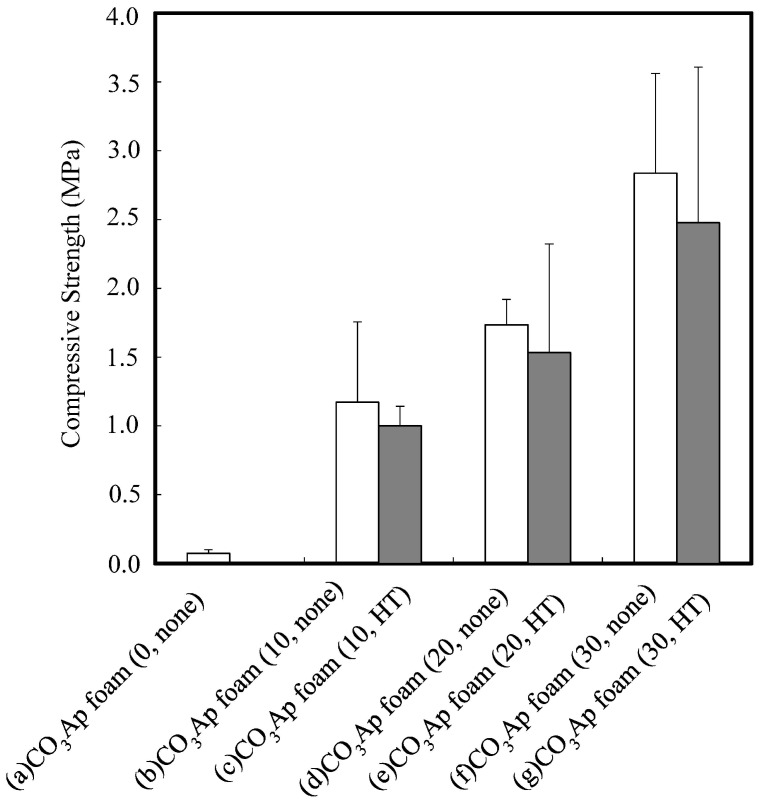
Compressive strength of CO_3_Ap foams: (**a**) CO_3_Ap foam (0, none); (**b**) CO_3_Ap foam (10, none); (**c**) CO_3_Ap foam (10, HT); (**d**) CO_3_Ap foam (20, none); (**e**) CO_3_Ap foam (20, HT); (**f**) CO_3_Ap foam (30, none); and (**g**) CO_3_Ap foam (30, HT) CO_3_Ap foam. Sample number in each group was five.

Figure 5 plots the compressive strength of the CO_3_Ap foam as a function of gelatin concentration, with and without heat treatment. Significant improvement (*p* < 0.05) in compressive strength was obtained in CO_3_Ap foams after gelatin reinforcement. No increase in the compressive strength was observed after heat treatment at 155 °C for 4 h. The average of the sample size was 15.0 mm × 15.0 mm × 11.0 mm.

**Figure 6:**
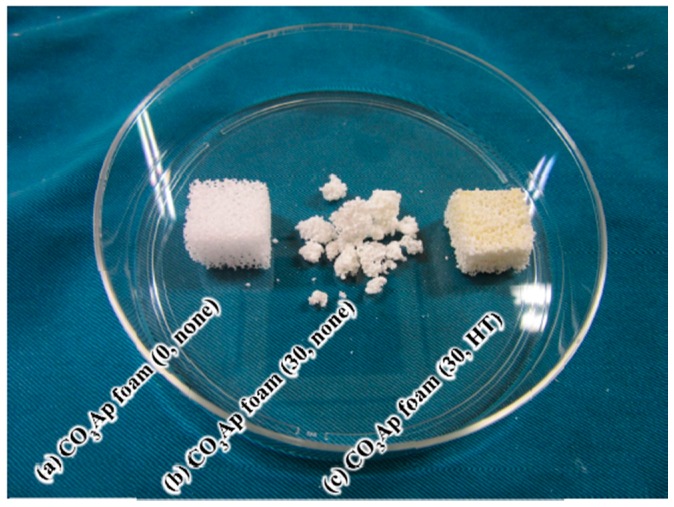
Photographs of CO_3_Ap foams when immersed in the saline solution at 37 °C for 24 h: (**a**) CO_3_Ap foam (0, none); (**b**) CO_3_Ap foam (30, none); and (**c**) CO_3_Ap foam (30, HT).

Figure 6 presents photographs typical of (a) CO_3_Ap foam (0, none); (b) CO_3_Ap foam (30, none); and (c) CO_3_Ap foam (30, HT) when immersed in the saline solution at 37 °C for 24 h. The gelatin-free CO_3_Ap foam maintained its shape in the saline solution. However, the gelatin-reinforced CO_3_Ap foam without heat treatment, or CO_3_Ap foam (30, none), collapsed and could not maintain its shape when similarly immersed. In contrast, the gelatin-coated CO_3_Ap foam with heat treatment, CO_3_Ap foam (30, HT), exhibited no appreciable change and retained almost the same shape as the original foam, even when immersed in the saline solution.

## 4. Discussion

The results obtained in this study clearly demonstrate that the reinforcing of CO_3_Ap foam with gelatin is a very effective way to improve its mechanical properties. The compressive strength increased from 34 kPa to approximately 3 MPa. In other words, the mechanical strength increased 100 times as a result of the gelatin coating. The compressive strength increased proportionally with the gelatin concentration. The amount of coated gelatin appeared to be proportional to the gelatin concentration. So, the mechanical strength of the CO_3_Ap foam was governed solely by the amount of gelatin; in other words, the mechanical strength of the gelatin-free CO_3_Ap foam was negligible.

Chemical crosslinking methods have been used to increase gelatin stability in aqueous media. Commonly used chemical crosslinkers include aldehydes (formaldehyde, glutaraldehyde, glyceraldehyde) [21], polyepoxy compounds [22] and carbodiimides [23]. The main limitation in the use of these products is that there is a possibility of the presence of some unreacted crosslinker inside the scaffold with consequent formation of toxic products during in vivo biodegradation [24]. For this reason, we chose heat treatment as the crosslinking method of gelatin in this study.

There was no difference before and after the heat treatment with respect to the mechanical strength of the CO_3_Ap foam, as demonstrated in Figure 5. Theoretically, the mechanical strength of gelatin should be increased by heat treatment because it results in crosslinking. This lack of difference may be partially due to the limited increase in the mechanical strength of gelatin resulting from crosslinking.

In contrast, a clear difference was observed in the behavior of the gelatin-reinforced CO_3_Ap foam with and without heat treatment, as demonstrated in Figure 6. The gelatin-free CO_3_Ap foam was stable, even when immersed in the saline solution, as observed in Figure 6a. However, the gelatin-reinforced CO_3_Ap foam could not maintain its shape when the foam had no heat treatment, as shown in Figure 6b. Gelatin swells in saline solution and its volume was expanded. Therefore, gelatin is thought to destroy CO_3_Ap foam. Non-crosslinked gelatin dissolves in saline, and thus, the gelatin-reinforced CO_3_Ap foam crumbled when immersed. The crosslinked gelatin-reinforced CO_3_Ap foam was stable in the saline solution, as shown in Figure 6c, and the marked difference in behavior is clearly caused by the presence of gelatin crosslinking. The gelatin did swell in saline even after heat treatment or crosslinking; however, because of the crosslinking, it did not collapse. Thus, the gelatin-coated CO_3_Ap foam became stable when the gelatin was crosslinked. The need for heat treatment appears to be idiosyncratic to gelatin-reinforced CO_3_Ap foam.

Enzymatically [25,26] or naturally [24,27] derived crosslinking agents might be other candidates of gelatin crosslinkers, apart from heat treatment, because of their low toxicity. Microbial transglutaminase (mTGase), derived from a variant of *Streptoverticillium mobaraense*, is a calcium-independent enzyme that catalyzes the formation of covalent cross-links between glutamine and lysine residues in proteins. This enzyme was reported to improve the mechanical properties of porous hydroxyapatite/collagen composites and gelatin-based scaffolds without cell cytotoxicity [25,26]. Genipin, the aglycone of geniposide (an iridoid glycoside isolated from the fruits of *Genipa americana* and *Gardenia* *jasminoides* Ellis), is a naturally occurring compound that can be used as a coupling agent for amino-containing materials [24]. Genipin was also reported to increase the stability of gelatin porous scaffold in aqueous media and improved its mechanical properties and it does not inhibit the osteoblast-like cell adhesion and proliferation [24].

Although no experiment was performed for the PLGA-coated and ε-caprolactone–coated CO_3_Ap foams, these polymers do not swell in saline solution, and therefore they would keep their shape. The need for heat treatment of these polymers is thought to be identical to that for gelatin-reinforced CO_3_Ap foam.

## 5. Conclusions

The mechanical strength of gelatin-coated CO_3_Ap foam increased proportionally with the increasing gelatin concentration and reached approximately 3 MPa when a 30 mass % gelatin solution was used for reinforcement. Heat treatment for cross-linking was the key to fabricating this foam as the absence of heat treatment resulted in the foam crumbling when immersed in a saline solution. As gelatin is known to exhibit good tissue response, based on these results, a further in vitro and in vivo study should be conducted to explore its potential.
